# Management of C5 Palsy After Anterior Cervical Decompression Using Oberlin Nerve Transfer: A Case Report

**DOI:** 10.7759/cureus.59217

**Published:** 2024-04-28

**Authors:** Antonio García-López, Javier Gutierrez-Pereira

**Affiliations:** 1 Orthopaedics and Traumatology, Hospital General Universitario de Alicante, Spanish National Reference Center for Brachial Plexus Surgery, Alicante, ESP

**Keywords:** oberlin procedure, nerve transfer, c5 palsy, cervical surgery, brachial plexus

## Abstract

C5 palsy is a potential complication of cervical decompression surgery from which many patients do not recover or partially recover function. We present the case of a 48-year-old patient who developed elbow flexion paralysis after anterior decompression surgery with fusion of the C5-C7 levels. Muscle function was not spontaneously restored until eight months after surgery. In this case, we performed an Oberlin procedure to restore the function of the arm. Muscle strength (5/5) and volume were obtained 13 months after surgery. A reasonable waiting period is required after C5 palsy in case spontaneous recovery occurs. Treatment decision should be based on the patient’s symptoms. Nerve transfers have been shown to be effective when performed after six months, especially in Oberlin transfer.

## Introduction

C5 palsy responds to a decrease of the deltoid and brachial biceps muscle strength in at least one level measured by manual muscle testing (MMT) and can be frequently observed in up to 6% of posterior decompression and 5% of anterior decompression surgeries [1]. Associated with the motor symptoms, the appearance of sensory deficits, numbness, and pain in the shoulder are common. The usual therapeutic approach after this complication is conservative management, usually adopted with optimism, as 41.5% of cases do not recover or partially recover with permanent consequences [2,3].

When there is function restoration, it occurs after 4.4 months on average, varying from three weeks to eight months. Foraminal decompression has been tried in some cases [4] with deficient results. Eskander et al. [5] suggested that spine rotation is a strong predictor and risk factor of this complication.

The indication for nerve repair surgery should not be delayed beyond eight months. The primary aim of the procedure is to restore the lost function. In these cases, the transfer of fascicles from the intact ulnar nerve to the musculocutaneous nerve, known as Oberlin transfer, is a solution to restore elbow flexion and spinal accessory to suprascapular nerve transfer to restore shoulder function.

## Case presentation

**Figure 1:**
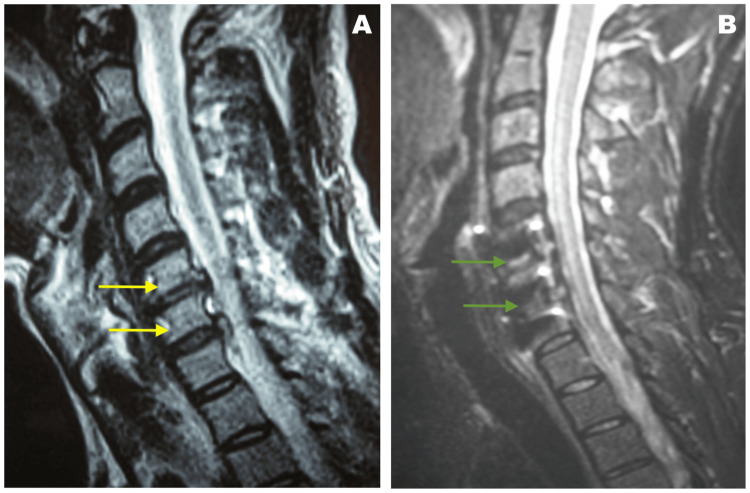
Magnetic resonance imaging findings. A: Preoperative imaging showing disc herniation (yellow arrows) at the C5-C6 and C6-C7 levels. B: Postoperative imaging after anterior decompression with the fusion of the affected levels (green arrows).

A 48-year-old male patient with a history of anterior decompression with a fusion of C5-C7 levels (Figure 1) developed paralysis of the scapulohumeral muscles, specifically abduction and rotations, and incapacity for elbow flexion right after surgery. The patient recovered shoulder mobility but biceps and brachialis muscle function were not restored after eight months of surgery with the examination based on MMT (Table 1).

**Table 1:** Exploration after cervical decompression surgery

Muscle	Power
Deltoid	4/5
Supraspinatus	4/5
Infraspinatus	4/5
Teres minor	4/5
Teres major	5/5
Subscapularis	5/5
Biceps brachii	0/5
Brachialis	0/5
Triceps	5/5
Brachioradialis	5/5

Surgical technique

**Figure 2:**
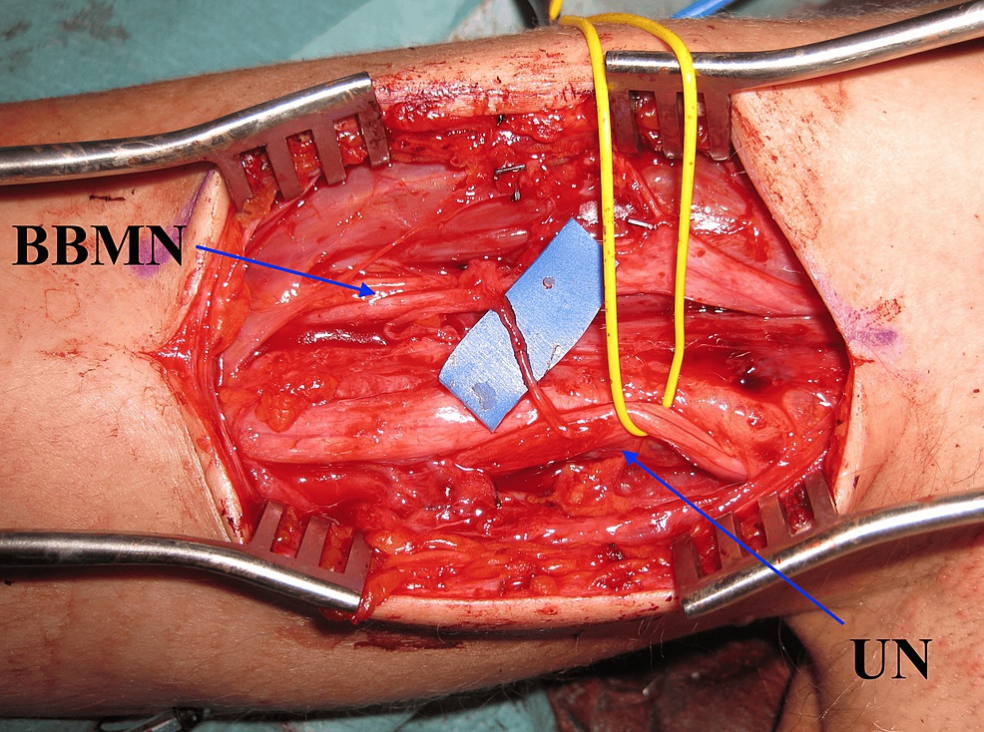
Oberlin procedure. The Oberlin procedure consists of performing a transfer of a fascicle of the ulnar nerve (yellow) with a predominant function of the flexor carpi ulnaris muscle to the motor branch of the musculocutaneous nerve. BBMC: biceps branch of musculocutaneous nerve; UN: ulnar nerve

**Figure 3:**
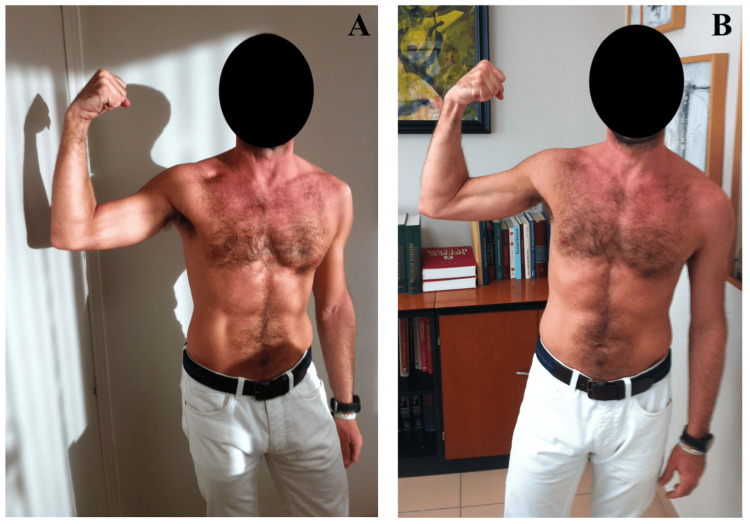
Postoperative aesthetic outcome. A, B: Postoperative aesthetic outcome showing the elbow flexion of the affected limb.

We performed a medial arm approach and a transfer of a nervous fascicle of the ulnar nerve with the predominant function of the flexor carpi ulnaris to the motor branch of the musculocutaneous nerve, which innervates the biceps brachii [6] (Figure 2). The initial contraction of the biceps muscle occurred six months after nerve transfer surgery. Total muscle strength and volume were achieved 13 months after surgery, with a balance of 4+/5 for the biceps brachii and brachialis (Figure 3).

The patient stated that for biceps contraction using strength, he needed to close his hand, which he did not consider a problem because most of the biceps contractions were done by picking up objects or fastening exercises requiring closing the hand. There was no loss of strength, mobility, or sensitive variations of his hands.

## Discussion

As many patients recover spontaneously after C5 palsy, a waiting period is required. This period ranges between three and four months to visualize the delimitations of the lesions and six to eight months to evaluate the state of recovery after core lesions [1,2].

Apart from spine rotation [5], other risk factors have been reported as multiple-level decompression [2,7], combined anterior and posterior cervical approach [8], performing a wide laminectomy, or spine deviation [9]. The initial ossification of the posterior longitudinal ligament or severe nerve injuries with <2 levels on the MMT are more unlikely to recover. Nevertheless, in female patients or cases with a lower level of decompression, the restoration seems to be total [8].

The reason why this pathology originates remains unknown. Some believe that palsy is caused by the direct lesion of the root, the instrumentation, or surgical manipulation [10], while others believe it originates from the shearing force of the spine on a well-anchored root in the foramen [11]. Hasegawa et al. proposed an initial neurological damage of the spine on the anterior horn [12]. However, some reasons explain why palsy originated after surgery.

In many cases, palsy has a good prognosis, with neurological and functional restoration. However, conservative treatment is adopted with too much optimism.

Physical therapy does not seem to be very effective [3]. Foraminal decompression has been attempted [4] in some cases with deficient results. Foraminotomy has been suggested to be a protective factor [11]. Miranda et al. [13] presented a video of a similar case with bilateral symptoms as a complication of posterior decompression.

Nerve transfers have been shown to be effective as in our case when performed after six months, especially in Oberlin transfer [6,14], consisting of the transfer of 15% of the ulnar nerve fascicles to the motor branches of the musculocutaneous nerve that innervate the biceps. The nerves used in this procedure have dispensable or redundant functions and are located near the terminal motor organ to be reinnervated, hence, there is no loss of function in the donor area. We propose this technique widely used in peripheral nerve surgery to be considered after C5 palsy caused by cervical decompression.

As a reasonable waiting period is required after any iatrogenic nerve injury, many surgeons prefer to wait for approximately six months to indicate repair surgery [1,2,14]. A too early indication of this procedure would ignore patients who could present a spontaneous recovery and a too late indication would affect the success of the procedure. In any case, treatment decisions should be based on the patient’s symptoms.

Our patient demonstrated an excellent nerve transfer outcome. He had a preoperative motor strength of biceps brachii 0/5 and brachialis 0/5 and a postoperative balance of 4+/5 for both biceps brachii and brachialis. The postoperative volume of the affected limb was very similar to that of the contralateral one.

## Conclusions

A reasonable waiting period is required after iatrogenic C5 palsy as in many cases spontaneous recovery occurs. We present the case of a patient who did not recover function eight months after cervical decompression for whom we performed an Oberlin-type nerve transfer with excellent results.
